# Networked neural spheroid by neuro-bundle mimicking nervous system created by topology effect

**DOI:** 10.1186/s13041-015-0109-y

**Published:** 2015-03-22

**Authors:** Gi Seok Jeong, Joon Young Chang, Ji Soo Park, Seung-A Lee, DoYeun Park, Junsung Woo, Heeyoung An, C Justin Lee, Sang-Hoon Lee

**Affiliations:** Department of Biomedical Engineering, College of Health Science, Korea University, Seoul, 136-100 South Korea; Center for Neural Science and WCI Center for Functional Connectomics, Korea Institute of Science and Technology (KIST), Seoul, 136-791 South Korea; Neuroscience Program, University of Science and Technology (UST), Daejeon, 305-350 South Korea; KU-KIST Graduate School of Converging Science and Technology, Korea University, Seoul, 136-701 South Korea

**Keywords:** Neurospheroid, Neural spheroid networking, Deep hemicylindrical channel, Neural bundle, Nerve-like structure

## Abstract

In most animals, the nervous system consists of the central nervous system (CNS) and the peripheral nervous system (PNS), the latter of which connects the CNS to all parts of the body. Damage and/or malfunction of the nervous system causes serious pathologies, including neurodegenerative disorders, spinal cord injury, and Alzheimer’s disease. Thus, not surprising, considerable research effort, both in vivo and in vitro, has been devoted to studying the nervous system and signal transmission through it. However, conventional in vitro cell culture systems do not enable control over diverse aspects of the neural microenvironment. Moreover, formation of certain nervous system growth patterns in vitro remains a challenge. In this study, we developed a deep hemispherical, microchannel-networked, concave array system and applied it to generate three-dimensional nerve-like neural bundles. The deep hemicylindrical channel network was easily fabricated by exploiting the meniscus induced by the surface tension of a liquid poly(dimethylsiloxane) (PDMS) prepolymer. Neurospheroids spontaneously aggregated in each deep concave microwell and were networked to neighboring spheroids through the deep hemicylindrical channel. Notably, two types of satellite spheroids also formed in deep hemispherical microchannels through self-aggregation and acted as an anchoring point to enhance formation of nerve-like networks with neighboring spheroids. During neural-network formation, neural progenitor cells successfully differentiated into glial and neuronal cells. These cells secreted laminin, forming an extracellular matrix around the host and satellite spheroids. Electrical stimuli were transmitted between networked neurospheroids in the resulting nerve-like neural bundle, as detected by imaging Ca^2+^ signals in responding cells.

**Electronic supplementary material:**

The online version of this article (doi:10.1186/s13041-015-0109-y) contains supplementary material, which is available to authorized users.

## Background

The nervous system in an animal transmits signals between each organ and the brain, serving to coordinate voluntary and involuntary activities [1-3]. In most animals, the nervous system consists of the central nervous system (CNS) and peripheral nervous system (PNS), the latter of which connects the CNS to all parts of the body [3-5]. Damage and/or malfunction of the nervous system causes serious pathologies, including neurodegenerative disorders, spinal cord injury, and Alzheimer’s disease. Given its prominent functional role, the nervous system has been the continuing focus of extensive studies.

One aspect of nervous system function that attracted considerable attention is signal transmission through the system. Signals within the nervous system are transmitted by an electrochemical wave called an action potential, which travels along the nerves, composed of cylindrical bundles of fibers consisting mostly of neural axons. The signal is transmitted between nerves by small amounts of neurotransmitter molecules released at nerve junctions, termed synapses. A variety of in vitro approaches have been developed in an attempt to understand the signal transduction mechanisms of this critically important system. However, conventional in vitro cell culture plates do not provide the ability to control diverse features of the neural microenvironment, and formation of certain neuronal growth patterns that mimic those that occur in vivo remains a challenge [6-8]. Recent progress in microfluidics, including micro contact printing and micro- and nano-topology fabrication technology, have allowed the culture of neuronal cells in a well-defined microenvironment, enabling the control of neuron and glial cell structuring processes. Microscale chemical [9,10] and topological patterns have proven invaluable for the study of neuronal behavior. Representative examples of these techniques include gradient control of soluble biochemical cues [11-14], micro-engineered grooved patterns [15,16], and biochemically modified grooved substrates [17,18]. These methods have been used extensively for guiding the growth of neurons [8,15,16,19] and promoting neuro-networking [7,20]. Although these approaches yield well-defined, networked neural cultures, it remains difficult to create a neural functionality close to that of the in vivo environment because cell culture conditions are restricted to a two-dimensional (2D) surface. Three-dimensional (3D) neuro-spheroid and -bundle formation of the nervous system by culturing on controlled microstructures, which have been shown to support successful growth of neurites, have been proposed as an alternative for mimicking the in vivo microenvironment [14,21,22].

Although 2D and 3D formation in in vitro nervous systems facilitate neurite growth and networking, most such models are based on the growth of single neurons or a single cell-cell network [7,8,13,18] using primary neuro progenitor cells. However, the nervous system in the animal is created by the growth of multiple cell types, including neurons and glia, which provide structural and metabolic support. In addition, conventional neural cell culture methods have a limited ability to mimic the connections of the nervous system between one part of the body and another through fiber bundles. To this end, several studies have been performed to fabricate three-dimensional (3D) neural networks using a microwell array [7,21-23]. Although, these studies demonstrated successful formation of spheroids and neural networking, it is still challenging to create a bundle-like neural networking formation which mimics the nervous system between spheroids. To address this limit, we have demonstrated that topological factors are critical for the formation of a nervous system, and further showed that a hemicylindrical channel is more effective in guiding neural outgrowth than a rectangular channel [24]. However, the neural bundle in this system was weakly connected through the hemicylindrical channel, and it was difficult to observe signal transmission through the bundle. For improved formation of bundle-like structures, we discovered that the channel barrier plays an important role in guiding well-defined outgrowth of multiple neurites, and quantitative studies for the barrier effect for the nervous system formation were required. In this study, we demonstrate a 3D nervous system model in which neurospheroids are networked to neighboring neurospheroids by a nerve-like network through deep hemicylindrical channels. To fabricate deep hemicylindrical channels, we introduced a method for extracting remnant poly(dimethylsiloxane) (PDMS) prepolymer from microwells and channels. The resulting concave wells supported the self-aggregation of host neurospheroids, and the deep hemicylindrical channel between concave wells provided excellent guidance of neural growth. Unlike the case for shallow hemicylindrical channels, satellite neurospheroids formed in the deep hemicylindrical channel. These satellite spheroids, guided by the deep hemicylindrical channel, played a bridging role in forming networks between host spheroids, enhancing the formation of strong nerve-like structures. To confirm this topological effect on the formation of nerve-like structures, we conducted a series of experiments comparing deep hemicylindrical channel well networking (HCWN) systems with shallow HCWN systems. These experiments demonstrated successful differentiation of neural progenitor cells to glia and neurons in the deep HCWN system. Moreover, subsequent Ca^2+^ imaging revealed propagation of electrical stimuli between networked host spheroids, confirming formation of functional nerve-like networks. Laminin membranes also developed around host and satellite neurospheroids during the process of neural progenitor cell differentiation. The proposed 3D nervous system could be extended to serve as a model for pathophysiological studies of the nervous system as well as nervous system diseases.

## Results

### Formation of deep HCWN features through PDMS surface tension

**Figure 1:**
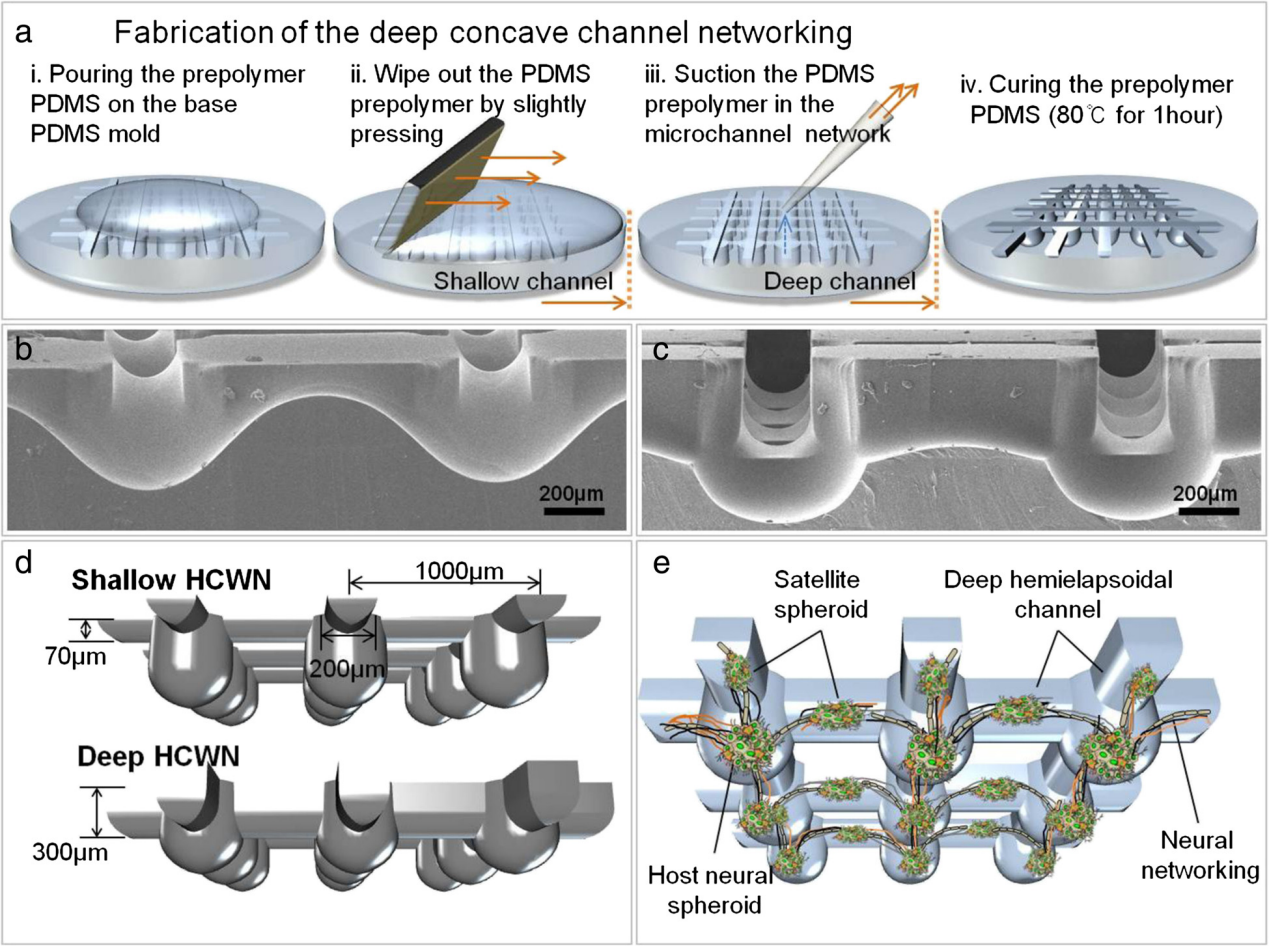
**Neural network formation in a deep HCWN system. (a)** Concave channel networks were fabricated by exploiting the surface tension of a PDMS prepolymer, as follows: i) Prepolymer PDMS was poured onto a base mold (PDMS). ii) The prepolymer was removed from the base mold by wiping out liquid PDMS, forming a meniscus. iii) For deep HCWN plates, PDMS prepolymer left behind after step ii is removed by suctioning. iv) After curing, the base mold was used for fabrication of a prepolymer PDMS concave channel network. **(b,c)** SEM images of a cross-section of the concave channel network, showing connections of concave well arrays with concave channels in shallow HCWN plates **(b)** and deep HCWN plates **(c)**. **(d)** Schematic view showing the dimensions of shallow and deep HCWN plates. **(e)** Schematic depiction of formation of a neural network, including satellite spheroids and neurite bundles, in a concave channel network.

Using a sweeping and suctioning process, we successfully fabricated shallow and deep hemicylindrical channels, and concave microwells from the PDMS base mold without the use of complicated devices or processes. For fabrication of shallow HCWN plates, only a sweeping process was employed, resulting in the generation of a shallow (*~*70 μm deep) hemicylindrical channel. The residual PDMS polymer was approximately 41.97 ± 3.57 mg (after sweeping) and 33.91 ± 2.75 mg (after suction) in the base mold (Additional file 1: Figure S1e). Figure 1b shows a side view of an SEM image of the shallow hemicylindrical channel and concave wells integrated in the HCWN system. Deep HCWN plates were prepared similarly, but with addition of a suctioning step to remove remnant PDMS polymer. After suctioning, the height of the hemicylindrical channel was almost the same as the rectangular channel height (~300 μm) of the PDMS base mold (Figure 1c). Figure 1d shows an illustration of the shape and dimensions of shallow and deep HCWN plates. As shown in the figures, the deeper hemicylindrical channel is suitable for generating satellite spheroids owing to the comparatively smooth joint of the deep channel (Figure 1e).

### Topological effects of shallow and deep HCWN systems on neural network formation

**Figure 2:**
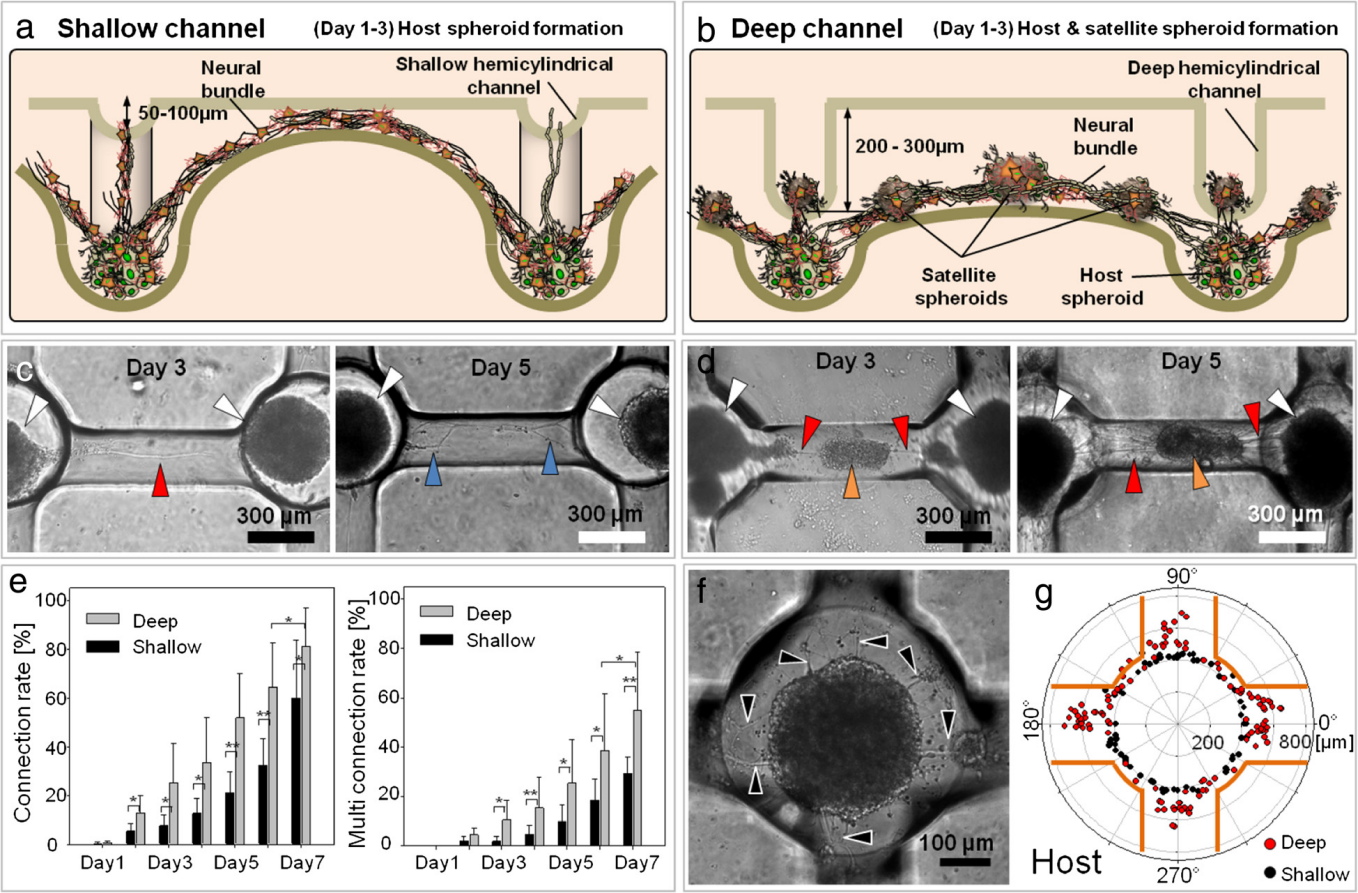
**Comparison of the effect of shape on neural network formation in shallow and deep HCWN systems. (a, b)** Schematic illustration of neural network formation on shallow and deep HCWN systems. **(c)** Optical microscope images of neural network formation in the shallow HCWN system. After neural cell seeding, host spheroids (white arrowheads, day 3) formed in concave wells, and neurite outgrowth from the host spheroid was observed in the shallow hemicylindrical channel (red arrowhead, day 3). Over time, network formation was observed between host spheroids in the shallow HCWN system (blue arrowheads, day 5). **(d)** Optical microscope images of neural network formation in the deep HCWN system. After neural cell seeding, host spheroids (white arrowheads, day 3) formed in concave wells, and satellite spheroids were simultaneously generated in the deep hemicylindrical channel (orange arrowheads, day 3). Over time, multi-neurite networks formed between host spheroids and satellite spheroids in deep hemicylindrical channels in the deep HCWN system (red arrowheads, day 5). **(e)** Connection rate of neurites between host spheroids in shallow and deep HCWN systems. Statistical analysis was performed using student’s *t*-test. Data shown are mean values (%) ± standard error with N=16 (connection rate measurements from four regions with 310 channels) for deep HCWN and N =15 (connection rate measurements from four region with 315 channels) for shallow HCWN (*P < 0.05; **P<0.01). At day 7, the total connection number was measured: 251 networks out of 310 deep HCWN and 110 networks out of 315 shallow HCWN. **(f)** Optical microscope image of neurite outgrowth from a host spheroid at day 5 (black arrowheads). **(g)** Direction and length of neurite outgrowth from a host spheroid at day 5 (red: deep (n=10), black: shallow (n=10)). Orange lines indicate the edges of the deep hemicylindrical channel.

To confirm the topological effect of the deep hemicylindrical channel in guiding neural cells, we performed comparison studies with shallow and deep HCWN systems. Networked neurospheroids were generated by first seeding both platforms with primary rat fetal cortical neural progenitor cells. The cells that did not settle in concave wells or hemicylindrical channels were removed by gentle pipetting (Additional file 1: Figure S3a). As a result, the seeded cells mainly remained in the concave microwells and deep hemicylindrical channels (Additional file 1: Figure S3b and c). We hypothesized that the residual cells in the deep hemicylindrical channel could play a critical role in fostering strong networking within neural bundles (Additional file 1: Figure S3c). Figure 2a and b show a schematic of the neural networking process in shallow and deep HCWN systems. As expected, neural cells in the shallow HCWN system only self-aggregated within the concave wells, forming neurospheroids (Figure 2c, white arrowheads); neural networks between spheroids formed via the shallow hemicylindrical channel (Figure 2c, red arrowheads), but satellite spheroids in channels were not observed. Neural cells seeded in deep HCWN plates also formed well-aggregated spheroids in concave microwells, but interestingly, the residual cells in the deep hemicylindrical channel generated satellite spheroids (Figure 2d, day 3, orange arrowheads). In the deep HCWN system, the neural network (red arrowheads) connected host (white arrowheads) and satellite (orange arrowheads) spheroids. About 5 days after seeding, neural networks between host and satellite spheroids were connected by multiple neurite strands growing along the deep hemicylindrical channel (Figure 2d, red arrowheads), with satellite spheroids acting as bridges during neural bundle formation (Figure 2b and d, red arrowheads). To quantify these results, we determined neural network connection rates, including overall rate and single and multiple connection rates, as defined in Additional file 1: Figure S4. These results, presented in Figure 2e, indicate that single, multiple, and bundle-formation connection rates were approximately 20% faster for the deep HCWN system (Figure 2e, left) at a given day in culture compared to the shallow HCWN system. About 80% of neurites were networked after 7 day in culture in the deep HCWN system. In contrast, approximately 60% of neurites were connected in the shallow HCWN system at this same time point (Figure 2e, left). To examine formation of nerve-like structures, we quantified multi-connections and bundles. Multi-connections in neural networks, defined as cases in which multiple connections or neural bundles formed along the channel (Additional file 1: Figure S4b), also increased over time. Seven days after cell seeding, the deep HCWN system achieved a multi-connection rate of about 60%; in contrast, the multi-connection rate in the shallow HCWN system at this time was about 30%. We further assessed neurite outgrowth from host spheroids in deep HCWN plates (Figure 2f), and quantified their length and direction of outgrowth (Figure 2g). Neurite outgrowth from host spheroids was mainly (~80%) connected along the hemicylindrical channel, indicating that the deep hemicylindrical channel has a strong affinity for neurites, providing guidance cues and directional control of neurite growth. In shallow HCWN plates, neurites frequently grew outside of the channel (Additional file 1: Figure S5).

### Topological effects of the deep HCWN system on satellite spheroid formation

**Figure 3:**
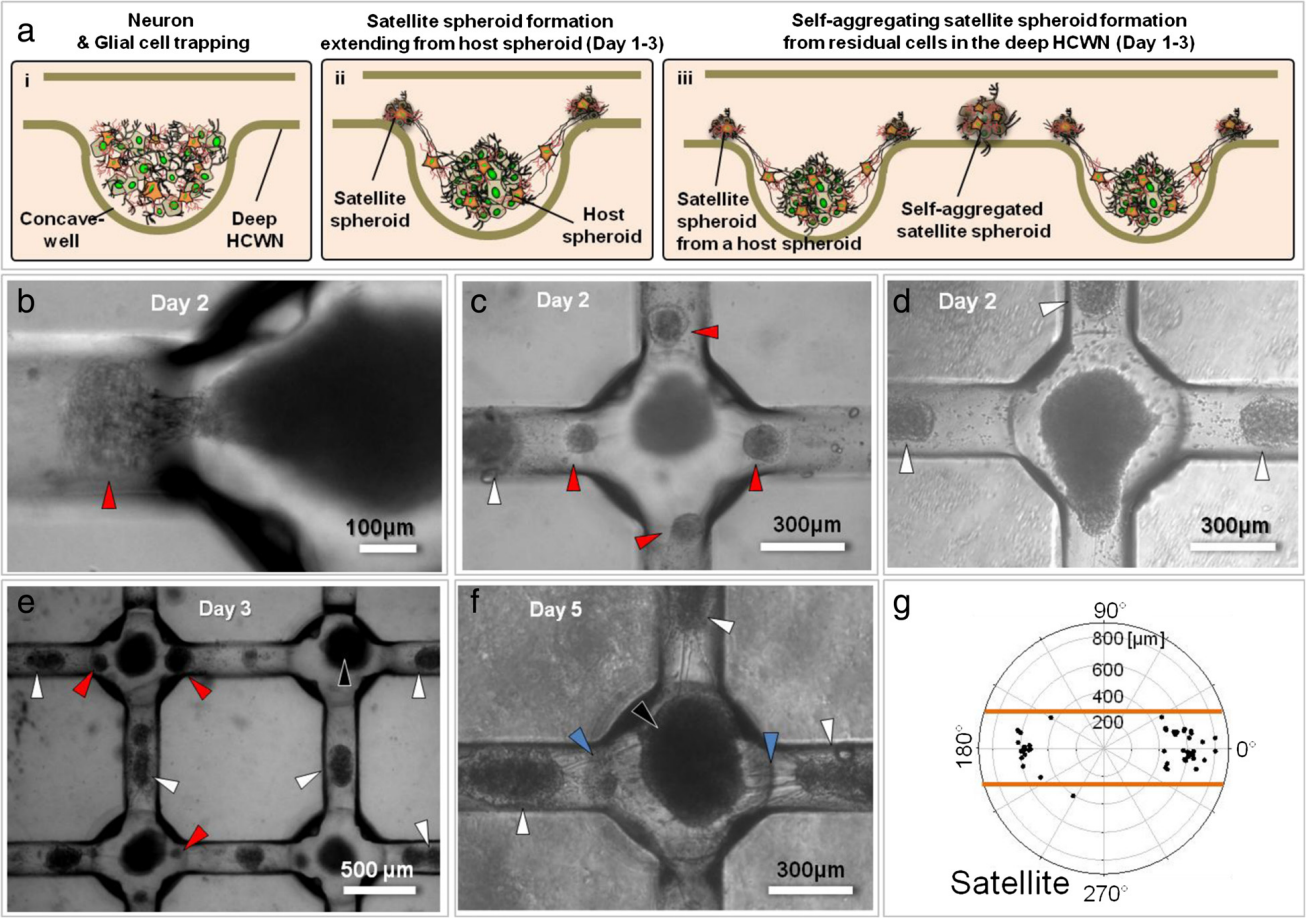
**Satellite spheroid formation in deep hemicylindrical channels. (a)** Schematic illustration of neural cell seeding (i) and satellite spheroid formation (ii-iii) on the deep HCWN system. Type I satellite spheroids are generated from the host spheroid (ii), whereas type II spheroids are generated by self-aggregation of residual cells (iii). **(b)** An optical microscope image of a satellite spheroid formed by migration from a host spheroid (type I, red arrowhead). **(c)** Satellite spheroids in the center of deep hemicylindrical channels spontaneously self-aggregated from residual cells (type II, white arrowheads). **(d)** Optical microscope image of type I and type II satellite spheroids formed simultaneously around the host spheroid on deep hemicylindrical channels. **(e)** Network formation between host spheroids (black arrowheads), type I spheroids (red arrowheads), and type II spheroids (white arrowheads) was observed in the deep HCWN system. **(f)** Multi-neurite connections between the host spheroid and satellite spheroids (blue arrowheads) in the deep HCWN system. **(g)** Direction and length of neurite outgrowth from satellite spheroids in a hemicylindrical channel at day 5. Orange lines indicate the edges of the deep hemicylindrical channel.

As noted above, satellite spheroids in the deep HCWN system enhanced neural network formation by serving an anchoring role between host spheroids. Our experiments showed two main types of satellite formation in the deep hemicylindrical channel, reflecting the nature of the satellite spheroid formation process. As shown in Figure 3a (i-iii), after the cell-seeding and wash-out step, neural cells settle down onto the concave microwells and deep hemicylindrical channel. One days after cell seeding, the host spheroids were self-aggregated in the concave microwell (host spheroid), and a number of neurite migrate out from the host spheroid, spontaneously generating type I satellite spheroids at the interfacing region between the concave microwells and hemispherical channel (Figure 3b and c, red arrowheads). Especially, residual cells in the deep hemicylindrical channel (Additional file 1: Figure S3c) were also self-aggregated to form type II satellite spheroids due to flat shape of the deep hemicylindrical channels (Figure 3c and d). Contrary to the shallow HCWN channel, these two types of spheroids, as well as host spheroids, are generated simultaneously throughout the deep HCWN system (Figure 3e). Furthermore, over time, these satellite spheroids merge with each other, contributing to the formation of multi-connections and bundles of nerve-like structures in the deep HCWN system (Figure 3f). Quantification of neurite outgrowth from satellite spheroids in the deep HCWN system (Figure 3g) showed that about 95% of neurites grew along the deep hemicylindrical channel, demonstrating that topology and satellite spheroids in the deep hemicylindrical channel effectively control neurite outgrowth.

### ECM membrane secretion and glial cell differentiation

**Figure 4:**
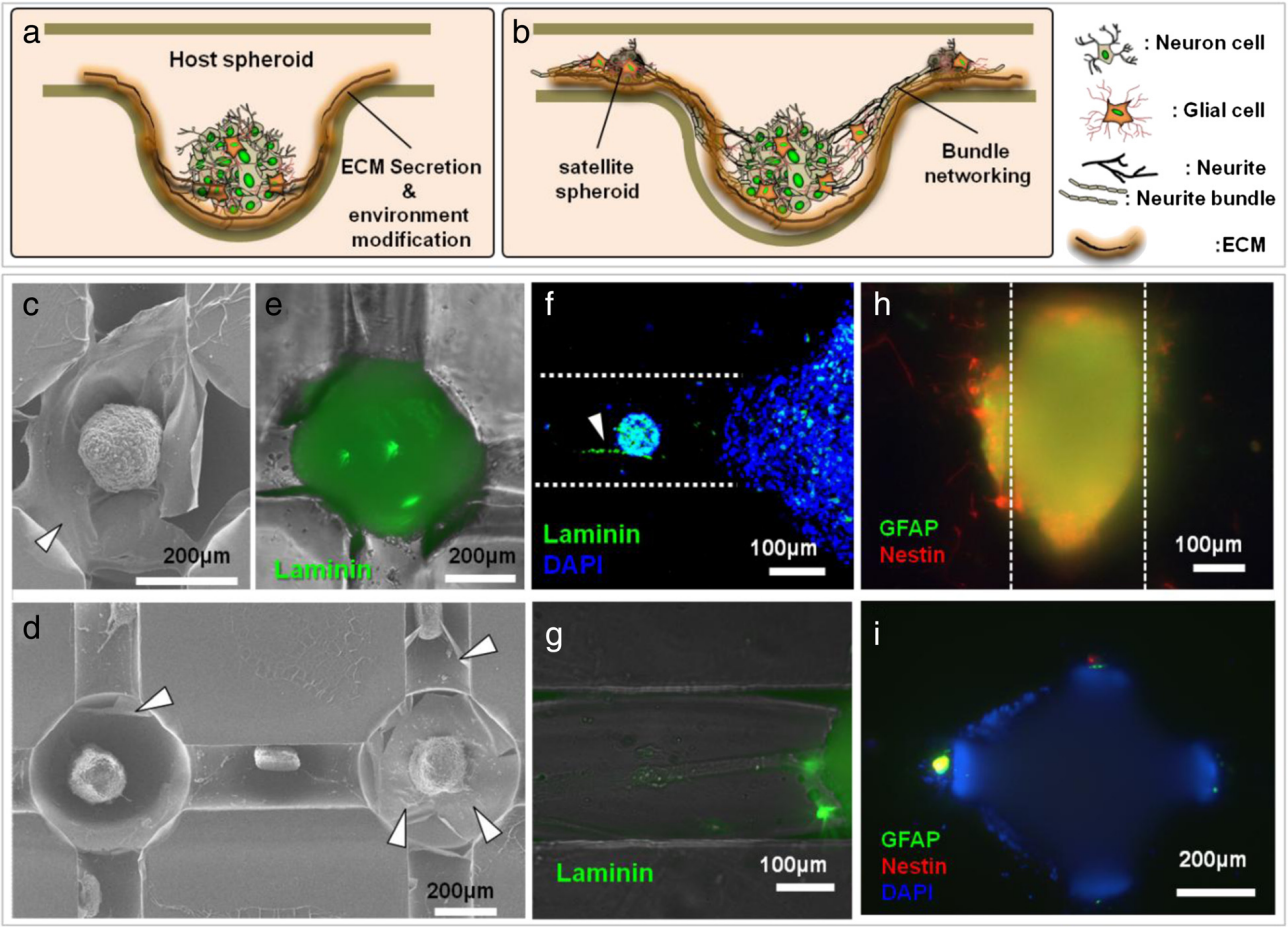
**ECM secretion and bundle formation within the neurospheroid networking in the deep HCWN system. (a**,**b)** Schematic depiction of ECM secretion from the spheroid **(a)**, and ECM secretion from the host and satellite spheroid and bundle formation during NSN formation **(b)**. **(c**,**d)** SEM images of the host spheroid and ECM membrane **(c)** and ECM formation around the spheroids **(d)**. **(e**,**f)** Merged image (fluorescence and optical microscope) of laminin **(e)** expression around a host spheroid. **(f)** Confocal microscope image of the area around a satellite and host spheroid. **(g)** Merged image (fluorescence and optical microscope) of laminin expression in a neurite bundle. **(h**,**i)** Fluorescence image of GFAP (green) and nestin (red) expression in satellite **(h)** and host **(i)** spheroids.

In this study, neural networking spheroids were generated by culturing cells without an ECM coating. However, it is well known that neural cells modify their environment by secreting the ECM component laminin [25]. Notably, ECM plays a pivotal role in neural cell guidance, proliferation, migration and differentiation, especially during the neuronal development process. We thus hypothesized that neural cells in the deep HCWN system secreted EMC, as depicted in the schematics shown in Figure 4a and b. Consistent with this possibility, SEM images revealed the presence of a membrane structure in deep HCWN plates 10 days after seeding primary neural cells, especially around host and satellite spheroids (Figure 4c and d). To verify that this membrane structure is ECM, we immuno-stained laminin. We found that laminin was strongly expressed around host spheroids (Figure 4e)z. Confocal and fluorescence microscope images of laminin expression in concave wells and hemicylindrical channels of deep HCWN plates showed that laminin fluorescence was highly expressed on host and satellite spheroids (Figure 4f and g; bright blue in merged images). Differentiation of progenitor neurons was assessed by immunostaining for the glial cell marker GFAP (glial fibrillary acidic protein) and progenitor cell marker nestin. Both markers were expressed in similar regions on satellite spheroids in the deep HCWN system (Figure 4h), particularly around the joint region of host spheroids (Figure 4i).

### Neuronal signal transmission through neurite bundles

**Figure 5:**
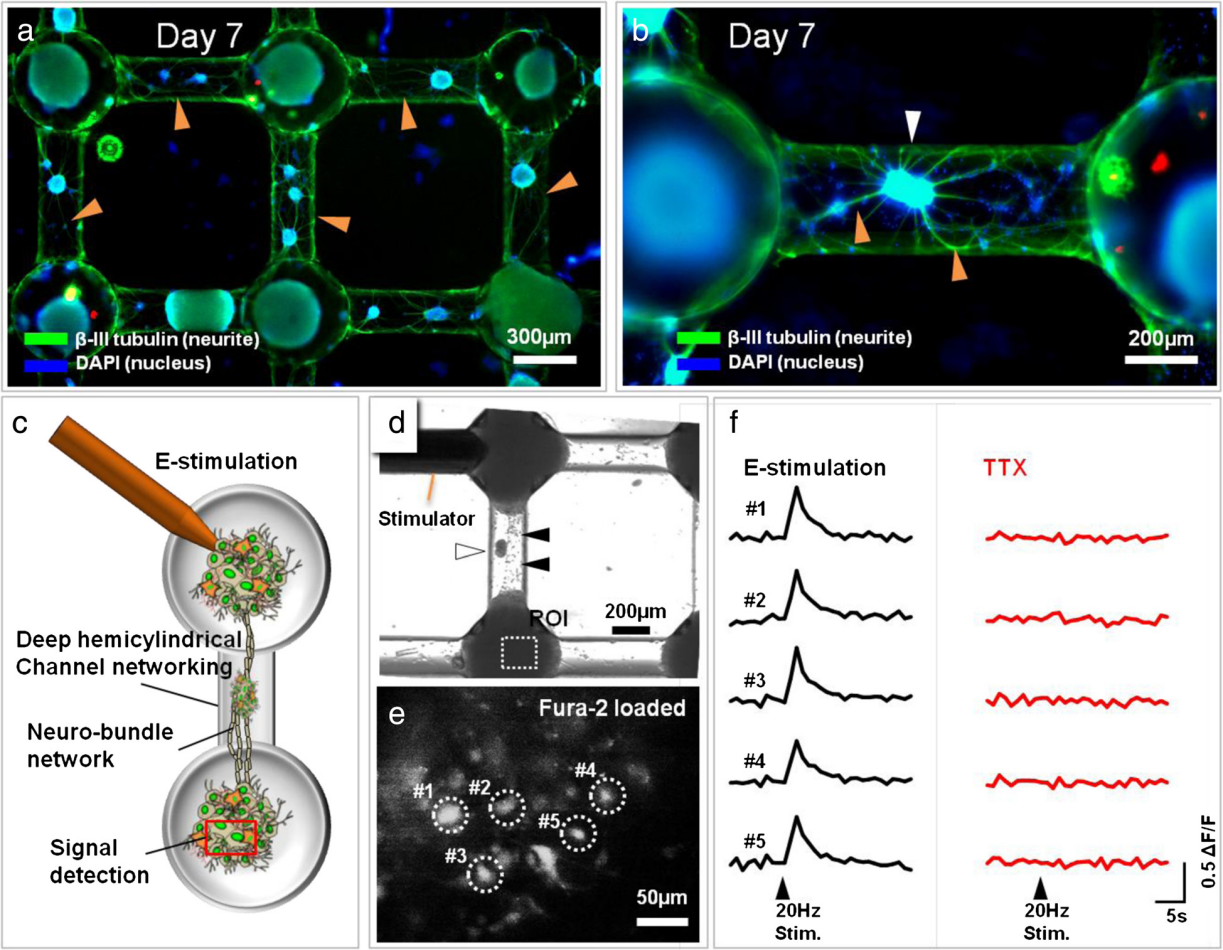
**Characterization of neural bundle-spheroid network function. (a)** Fluorescence image of neuronal spheroids. Green and blue indicate neurites (β-tubulin) and nuclei (DAPI), respectively. **(b)** Bundle formation in neurospheroid networks; each neural bundle is composed of several neurite fibers. **(c)** Schematic depiction of the method used to assess signal transmission within a neurospheroid network. One neuronal spheroid was electrically stimulated and the electrical signal was detected by imaging Ca2+ in another spheroid. Red rectangle indicates the region of interest (ROI). **(d)** An optical microscope image of an electric stimulator on the nerve-like bundle and the ROI at which Ca2+ signals were recorded. Black arrowheads indicate a neural bundle. **(e)** A fluorescence microscope image of Ca2+ signals (dotted circles) detected in the ROI shown in **(d)**. The white and black arrowheads indicate satellite spheroid and bundle formation in deep HCWN **(f)** Electrical transmission through the nerve-like bundle without (left) and with (right) TTX. Signals were detected at all sites prior to TTX treatment, and all signals were eliminated by TTX.

Neural cells were cultured in deep HCWN plates for 7 days to allow the development of neural networks connected through nerve-like bundles (Figure 5a and b). Satellite spheroids (Figure 5b, white arrowheads) in the deep hemicylindrical channel were anchored at the center of the channel and were connected to the host spheroids (Figure 5a and b, orange arrowheads), showing that satellite spheroids act as intermediaries to enhance structural networking between host spheroids. To determine whether this nerve-like network supports signal transmission functions, we applied an electrical stimulus to one spheroid (Figure 5c) and recorded Ca^2+^ responses of fura-2-loaded cells in a connected spheroid. As shown in Figure 5d, electrical stimulation at one point produced simultaneous Ca^2+^ fluorescence signals in responding cells located at a distance of ~1 mm (Figure 5d, white dotted rectangle) from the stimulator, which is indicative of transmission of a Ca^2+^ signal through a neural bundle (Figure 5e, white dotted circles) and confirming that spheroids are functionally networked. The stimulation-evoked Ca^2+^ response was transient and was abolished by the voltage-gated Na^+^ channel inhibitor tetrodotoxin (TTX; 0.5 μM), indicating that a stimulation-induced action potential could be propagated through the networked bundles, leading to a postsynaptic Ca^2+^ response at the recording site (Figure 5f). These results show that neural network bundles consist of several neuronal cells and connect spheroid to spheroid.

## Discussion

In a previous study, we compared neural network formation in a shallow hemicylindrical channel to that in a rectangular channel [24]. Although the nerve-like network in the shallow hemicylindrical microchannel was generated randomly and was weak, we found that the hemicylindrical channel provided suitable conditions for neurite outgrowth and networking. In the current study, we compared nervous system formation in shallow and deep hemicylindrical channels to observe the barrier effect. Our results demonstrated that the deep hemicylindrical channel exerts a strong topological influence over the generation of satellite neurospheroids, enhancing guidance of neurite outgrowth and creation of a bundle-like nerve system. Cells tended to aggregate at the center of the deep microchannel (Figure 2d, orange arrowheads) forming type 2 spheroids as the shape of the deep channels is flat and presents a high barrier which prevents neuronal outgrowth. The type 1 spheroids generally appeared at the smooth interfacing area between the concave well and hemicylindrical channel (Figure 3b, c, and e, red arrowheads). These two types of satellite spheroids act as anchors to enhance neural network formation in the deep HCWN system. Although further studies of satellite spheroids are needed, such behavior of neural cells in deep HCWN plates seems to be closely related with the topology of the channel. Despite seeding cells on untreated surfaces of deep HCWN plates, neurites grew along the center of the hemicylindrical microchannels even without ECM treatment. These results show that the curvature and depth of the HCWN channels provide a topology which is advantageous for forming a well-guided network of nerves.

Interestingly, the GFAP and Nestin were expressed around the satellite spheroid and at the joint region around the host spheroid, whereas they were not expressed in the host spheroid (Figure 4 h and i). This figure suggests that some progenitor cells around satellite spheroids and at the joint region are differentiating into glial cells, while cells in host spheroids do not differentiate into glia cells. Although further study is required, this phenomenon seems to reflect the glia cells’ role during the nervous system development stage.

Microenvironments have important role for central nerve system (CNS) development and neural stem cell (NSC) differentiation [25], and several ECM molecules have been identified that regulate cell growth, migration and proliferation [5,16], as well as differentiation of neural progenitor cells [6,8]. Although, microstructures and biochemical materials can provide cues for growth and differentiation of neuro-progenitor cells, it is still unclear how the neuro-progenitor cells modify the environment to enhance their growth and differentiation. In this study, the deep HCWN seems to enhance the formation of host and satellite spheroids that contributed by secreting laminin, which is well known for playing an important role in neural cell outgrowth and the differentiation of neural progenitor cells [25], and to induce differentiation of the neural cells without any pretreatment of biochemical attractants (Figure 4c, d, and e). The self-modified environment by the secretion of laminin in the deep HCWN can facilitate the formation of well-organized neuro-bundles.

## Conclusions

Using the deep HCWN system, we successfully developed a neural network connecting host and satellite neurospheroids. The host and satellite spheroids created at the deep HCWN structures through self-modification of the surrounding ECM environment. During network formation on the deep hemicylindrical channel network, neural progenitor cells successfully differentiated into glial and neuron cells, forming a laminin-containing scaffold around the host and satellite neurospheroids during nerve-like formation. Functional connectivity within the fabricated nerve-like network was demonstrated by monitoring transmission of an electrical stimulus using Ca^2+^ imaging. Furthermore, we found that satellite neurospheroids formed around the host neurospheroid on the deep hemicylindrical channel and in the center of the channel. These satellite neurospheroids act as anchoring points in the networks to play an important role in enhancing neural network formation. We expect that the proposed method will be extensively used as a brain or nervous system model for the drug screening and the physiological study.

## Methods

### Fabrication of master molds for deep and shallow HCWN systems

Shallow and deep HCWN plates consist of concave microwell arrays connected by a shallow (50–100 μm) or deep (*~*300 μm) hemicylindrical channel. HCWN plates were fabricated by first preparing a two-layered PDMS base mold consisting of cylindrical microwell arrays connected via a rectangular channel using a standard soft lithography process (Additional file 1: Figure S1a). Shallow HCWN plates were prepared by pouring PDMS prepolymer on the PDMS base mold (Figure 1a (i-ii) and Additional file 1: Figure S1b), and sweeping out PDMS prepolymer by lightly pressing the soft PDMS base mold using a glass slide (76 × 52 × 1.2 mm), as previously reported [24,26-28] (Figure 1a and Additional file 1: Figure S1b). For deep HCWN plates, the remnant PDMS prepolymer in microwells and channels was aspirated using a syringe (Figure 1a (i-iii) and Additional file 1: Figure S1c), leaving a hemicylindrical channel with a depth of approximately 300 μm–almost the same depth as the rectangular channel of the PDMS base mold. The residual PDMS prepolymer was measured using a micro balance (PAG214C, OHAUS, USA). For the measurement, the weight of PDMS base mold was measured first. The weight of PDMS base mold after prepolymer sweeping and suction was measured. By subtraction of weight of PDMS base mold, we measured the weight of remaining prepolymer after sweeping and suction. The residual PDMS prepolymer in the microwell and rectangular channel adopted a curved meniscus through surface tension, forming the deep hemicylindrical channels and concave wells. The PDMS prepolymer-formed meniscus was solidified by thermal curing on a hot plate (80°C for 1 hour) (Figure 1a (iv), and Additional file 1: Figure S1b and c (iv)). After fabrication, shallow and deep HCWN plates were replicated using SU-8 (Micro-Chem, Newton, MA, USA), and a convex SU-8 master mold was created (Additional file 1: Figure S1d and S2). Deep and shallow HCWN plates were ultimately fabricated by replicating the master mold with PDMS. The dimensions of shallow and deep HCWN systems are depicted in Figure 1d. For the deep HCWN system, the dimensions of cylindrical wells were 500 × 500 μm (diameter and depth); rectangular channels were 1 mm (length) × 200 μm (width) × 300 μm (depth) (Additional file 1: Figure S1b and c).

### Preparations of neural progenitor cells

Primary neural progenitor cells were isolated from a cortical region of prenatal (embryonic day 16) rats (DBL, Incheon, South Korea), and were collected by centrifugation at 10,000 rpm for 5 minutes [23,29,30]. After collection, progenitor cells were seeded in wells of deep and shallow HCWN plates and cultured in Neurobasal media (Gibco, Lifetechnonogies, NY, USA) containing B-27 Supplement (Gibco, Lifetechnonogies, NY, USA), 0.5 mM L-glutamine, and 1% of an antibiotics solution containing 10,000 units penicillin (Gibco, Lifetechnonogies, NY, USA) and streptomycin. All procedures conformed to the standards of the Institutional Review Board of Korea University.

### Cell culturing on shallow and deep HCWN plates

To seed a uniform number of cells into each concave well, we directly dropped 1 ml of a cell suspension (2.0 × 10^7^ cells/ml) on top of each type of HCWN plate followed by repeated gentle pipetting [31,32]. When cells had settled into concave well arrays and channels (10 minutes after seeding), culture medium was gently applied to remove cells that had not settled (Additional file 1: Figure S3). The medium was replaced with fresh medium every other day.

### Ca^2+^ imaging

Signal transmission between spheroids within formed neural networks was monitored by imaging Ca^2+^ in responding cells using the ratiometric fluorescent dye, Fura-2-AM (100 μM; loading time, 30 minutes). To test whether this Ca^2+^ response was evoked by neuronal action potential activity, we used tetrodotoxin (TTX), a voltage gated Na^+^ channel inhibitor. To block the neuronal activity, we treated the cells with TTX for at least 5 minutes using a bath application (ACSF recording solution containing TTX). Then, Ca^2+^ responses were measured upon electrical stimulation. In the presence of TTX, we could not see the Ca2+ response. The experimental details are now included in the method section. Cells were imaged by exciting at wavelengths of 340 and 380 nm, and collecting fluorescence at 510 nm using a CCD camera. Ca^2+^ concentration was determined by performing ratio calculations using Axon Imaging Workbench version 6.2 (Axon Instruments, Molecular Devices, Sunnyvale, CA, USA). To induce the neuronal activity-dependent Ca^2+^ responses in cells, we used the 20 Hz electrical stimulation, a subthreshold stimulation. This stimulation has been widely used to induce the neuronal activity in brain slice without causing long-term potentiation [33]. Cells in spheres were electrically stimulated with a 20-Hz, 1-second pulse, and images were acquired at a rate of 1 images/s. The relative change in Ca^2+^ concentration was normalized to baseline concentration, obtained by averaging 20 images prior to stimulation.

### Immunochemistry

For immunostaining, networked neurospheroids were first fixed for 20 minutes with 4% formaldehyde at 4°C, after which cells were permeabilized by incubating with phosphate-buffered saline (PBS) containing 0.1% Triton X-100 for 20 minutes at room temperature, blocked with PBS containing 3% bovine serum albumen (BSA) for 30 minutes, and then incubated with primary antibody overnight at 4°C. Neurite outgrowth was examined using a primary rabbit polyclonal antibody against the neurofilament, β-III tubulin (1:1000; Santa Cruz Biotechnology, Dallas, TX, USA), a marker of neurites. Astrocytes and neural progenitor/stem cells were examined using a primary rabbit polyclonal IgG antibody against GFAP (1:100; Santa Cruz Biotechnology, Dallas, TX, USA) and a primary anti-rat antibody against nestin (1:1000; Stemcell technologies, USA), respectively. After incubating overnight, cells were washed with PBS containing 0.1% BSA for 5 minutes, and then incubated with secondary antibodies (1:1000; Invitrogen, Carlsbad, CA, USA) for 1.5 hours at room temperature. Cells were washed again with PBS/0.1% BSA, and images were acquired with a fluorescence microscope (EVOS; Advanced Microscopy Group, Mills Creek, WA, USA) after counterstaining with 4,6-diamidino-2-phenylindole dihydrochloride (DAPI; Invitrogen).

For confirmation of laminin, day-10 networked neurospheroids on deep HCWN plates were fixed with 4% paraformaldehyde for 20 minutes at 4°C and permeabilized by incubating with PBS/0.1% Triton X-100 for 20 minutes at room temperature. Non-specific protein adsorption to cells and membranes of deep HCWN plates was blocked by incubating with PBS/0.1% BSA for 30 minutes at 4°C. Cells were then probed with anti-laminin (Abcam, Cambridge, UK) overnight at 4°C. Cells and membranes in deep HCWN plates were gently washed again with PBS/0.1% BSA and incubated with the appropriate Alexa Fluor 488- or 594- secondary antibody (Invitrogen) for 90 minutes at 4°C. Fluorescence images were obtained using a confocal laser-scanning microscope (Olympus, Japan).

### Scanning electron microscopy

Concave channel network shape, extracellular matrix (ECM) membrane, and neurospheroid-integrated neural networks in the deep HCWN system were analyzed by field emission-scanning electron microscopy (FE-SEM) using a JEOL 4701 F system (JEOL Ltd., Tokyo, JAPAN). Neural networks cultured in deep or shallow HCWN plates were first fixed with 2.5% glutaraldehyde in deionized water for 1 hour, then gently washed with deionized water, and subjected to secondary fixation in 1% osmium tetroxide in deionized water for 1 hour. Fixed concave-channel neural networks were dehydrated with a series of graded ethanol (25%, 50%, 75%, 95%, and 100%), then incubated with tert-butyl alcohol at room temperature for 30 minutes (three times) and frozen at −70°C. Neural networks on deep HCWN plates were freeze-dried until the tert-butyl alcohol had evaporated, then were mounted on a specimen stub with graphite tape, coated with palladium alloy, and observed by FE-SEM.

### Neurite connection and outgrowth measurements and statistical analysis

The direction and lengths of neurite outgrowths from host and satellite spheroids were measured in optical microscope images by image analysis using ImageJ (http://rsbweb.nih.gov/ij/). Neurite connections were manually determined from optical microscope images (Additional file 1: Figure S4). The statistical analysis was implemented using SPSS ver.12 (Chicago, IL, USA).

## Additional file

Additional file 1.
**Fabrication process and dimensions of shallow and deep HCWN plates.** (a) Fabrication of the PDMS base mold. (b) Process for fabricating shallow HCWN plates. (i) The PDMS prepolymer is poured onto the channel. (ii) The PDMS prepolymer is swept out by gently pressing the soft PDMS base mold using a glass slide. (iii) The PDMS prepolymer is cured at 80°C for 1 hour. (c) Process for fabricating deep HCWN plates. After performing steps (i) and (ii) as for the shallow system, the residual PDMS prepolymer was removed by suction (iii). (iv) After formation of deep HCWN system features, the PDMS plate was cured. (d) Picture of the deep HCWN (e) The residual PDMS prepolymer after sweeping (b) and suction (c). Statistical analysis was performed using student’s *t*-test. Data shown are mean values (%)±standard error with N=6 (individual connection rate measurements from four regions) (**P < 0.001). **Figure S2.** Schematic diagram of the SU-8 replica fabrication process for shallow and deep HCWN plates. 1. Fabrication of shallow and deep HCWN plates. 2. Pouring SU-8 n shall and deep HCWN plates. 3. Su-8 replica of the convex structure network. **Figure S3.** Cell seeding on shallow and deep HCWN plates. (a) A 1-ml cell suspension was directly seeded onto shallow and deep HCWN plates with repeated gentle pipetting. Ten minutes after seeding, neural cells had settled within the plate (i); culture medium was then gently applied to remove cells that did not settle (ii). (b, c) Settled cells after the wash-out process in the shallow HCWN system (b) and deep HCWN system (c). **Figure S4.** Definition of neurite connection. (a) Single neurite connection through the channel. (b) Multi-connection and bundled neurite networks through the channel. **Figure S5.** Neurites growing outside the channel in the shallow HCWN system.
