# Correction to: Networked neural spheroid by neuro-bundle mimicking nervous system created by topology effect

**DOI:** 10.1186/s13041-021-00797-4

**Published:** 2021-06-17

**Authors:** Gi Seok Jeong, Joon Young Chang, Ji Soo Park, Seung-A Lee, DoYeun Park, Junsung Woo, Heeyoung An, C. Justin Lee, Sang-Hoon Lee

**Affiliations:** 1 Department of Biomedical Engineering, College of Health Science, Korea University, Seoul, 136-100 South Korea; 2 Center for Neural Science and WCI Center for Functional Connectomics, Korea Institute of Science and Technology (KIST), Seoul, 136-791 South Korea; 3 Neuroscience Program, University of Science and Technology (UST), Daejeon, 305-350 South Korea; 4 KU-KIST Graduate School of Converging Science and Technology, Korea University, Seoul, 136-701 South Korea

## Correction to: Molecular Brain (2015) 8:17 https://doi.org/10.1186/s13041-015-0109-y

Following publication of the original article [1], the authors would like to correct the affiliations assigned to the author ‘Heeyoung An’. Originally, only affiliation 2 was assigned to this author, while the author is affiliated to both affiliations 2 and 4:

2 Center for Neural Science and WCI Center for Functional Connectomics, Korea Institute of Science and Technology (KIST), Seoul 136-791, South Korea.

4 KU-KIST Graduate School of Converging Science and Technology, Korea University, Seoul 136-701, South Korea.

The author list has been updated in this Correction article and the original article [1] has been corrected.

## References

[1] Jeong GS, Chang JY, Park JS, Le SA, Park D, Woo J, An H, Lee CJ, Lee S-H (2015). Networked neural spheroid by neuro-bundle mimicking nervous system created by topology effect. Mol Brain.

