# Supplementary material for: Networked neural spheroid by neuro-bundle mimicking nervous system created by topology effect
Source: Mol Brain. 2015 Mar 22;8:17. doi: 10.1186/s13041-015-0109-y (PMC4379946; doi:10.1186/s13041-015-0109-y)
Supplement: Additional file 1. — Fabrication process and dimensions of shallow and deep HCWN plates. (a) Fabrication of the PDMS base mold. (b) Process for fabricating shallow HCWN plates. (i) The PDMS prepolymer is poured onto the channel. (ii) The PDMS prepolymer is swept out by gently pressing the soft PDMS base mold using a glass slide. (iii) The PDMS prepolymer is cured at 80°C for 1 hour. (c) Process for fabricating deep HCWN plates. After performing steps (i) and (ii) as for the shallow system, the residual PDMS prepolymer was removed by suction (iii). (iv) After formation of deep HCWN system features, the PDMS plate was cured. (d) Picture of the deep HCWN (e) The residual PDMS prepolymer after sweeping (b) and suction (c). Statistical analysis was performed using student’s t-test. Data shown are mean values (%)±standard error with N=6 (individual connection rate measurements from four regions) (**P < 0.001). Figure S2. Schematic diagram of the SU-8 replica fabrication process for shallow and deep HCWN plates. 1. Fabrication of shallow and deep HCWN plates. 2. Pouring SU-8 n shall and deep HCWN plates. 3. Su-8 replica of the convex structure network. Figure S3. Cell seeding on shallow and deep HCWN plates. (a) A 1-ml cell suspension was directly seeded onto shallow and deep HCWN plates with repeated gentle pipetting. Ten minutes after seeding, neural cells had settled within the plate (i); culture medium was then gently applied to remove cells that did not settle (ii). (b, c) Settled cells after the wash-out process in the shallow HCWN system (b) and deep HCWN system (c). Figure S4. Definition of neurite connection. (a) Single neurite connection through the channel. (b) Multi-connection and bundled neurite networks through the channel. Figure S5. Neurites growing outside the channel in the shallow HCWN system. [file 13041_2015_109_MOESM1_ESM.pdf]

Supplementary Figures

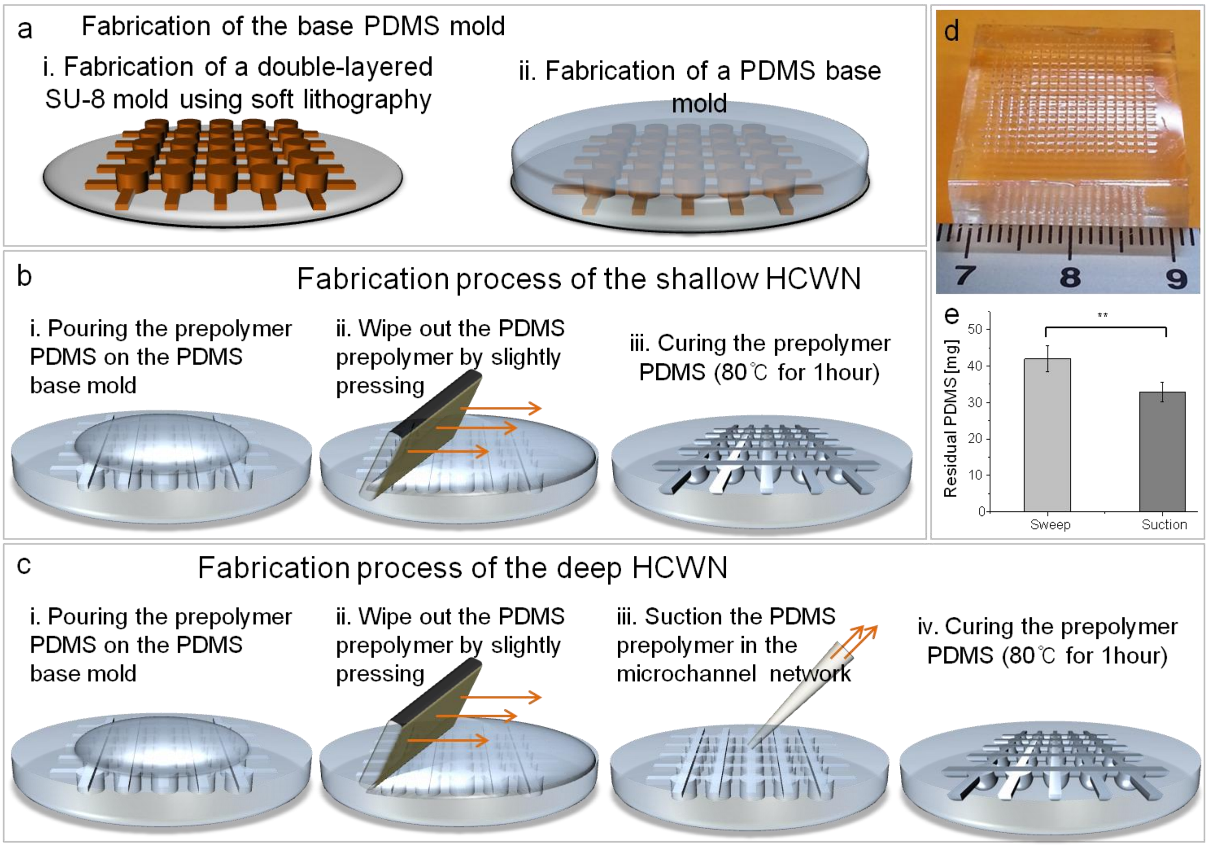

Fig. S1. Fabrication process and dimensions of shallow and deep HCWN plates. (a) Fabrication of the PDMS base mold. For base channel fabrication, PDMS prepolymer is poured onto a two-layered SU-8 mold and then cured (i-ii). (b) Process for fabricating shallow HCWN plates. (i) After detaching the PDMS base mold, the PDMS prepolymer is poured onto the channel. (ii) After degassing, the PDMS prepolymer is swept out by gently pressing the soft PDMS base mold using a glass slide (76 x 52 x 1.2 mm). Surface tension creates PDMS meniscuses corresponding to nascent cylindrical microwells and shallow hemicylindrical channels. (iii) The PDMS prepolymer is cured at 80°C for 1 hour. (c) Process for fabricating deep HCWN plates. After performing steps (i) and (ii) as for the shallow system, the residual PDMS prepolymer was removed by suctioning (iii), resulting in the formation of deep hemicylindrical channels with concave microwells through surface tension. (iv) After formation of deep HCWN system features, the PDMS plate was cured. (d) Picture of the deep HCWN (e) The residual PDMS prepolymer after sweeping (b) and suction (c). Statistical analysis was performed using student's t-test. Data shown are mean values (%)±standard error with N=6 (individual connection rate measurements from four regions) (**$P < 0.001$).

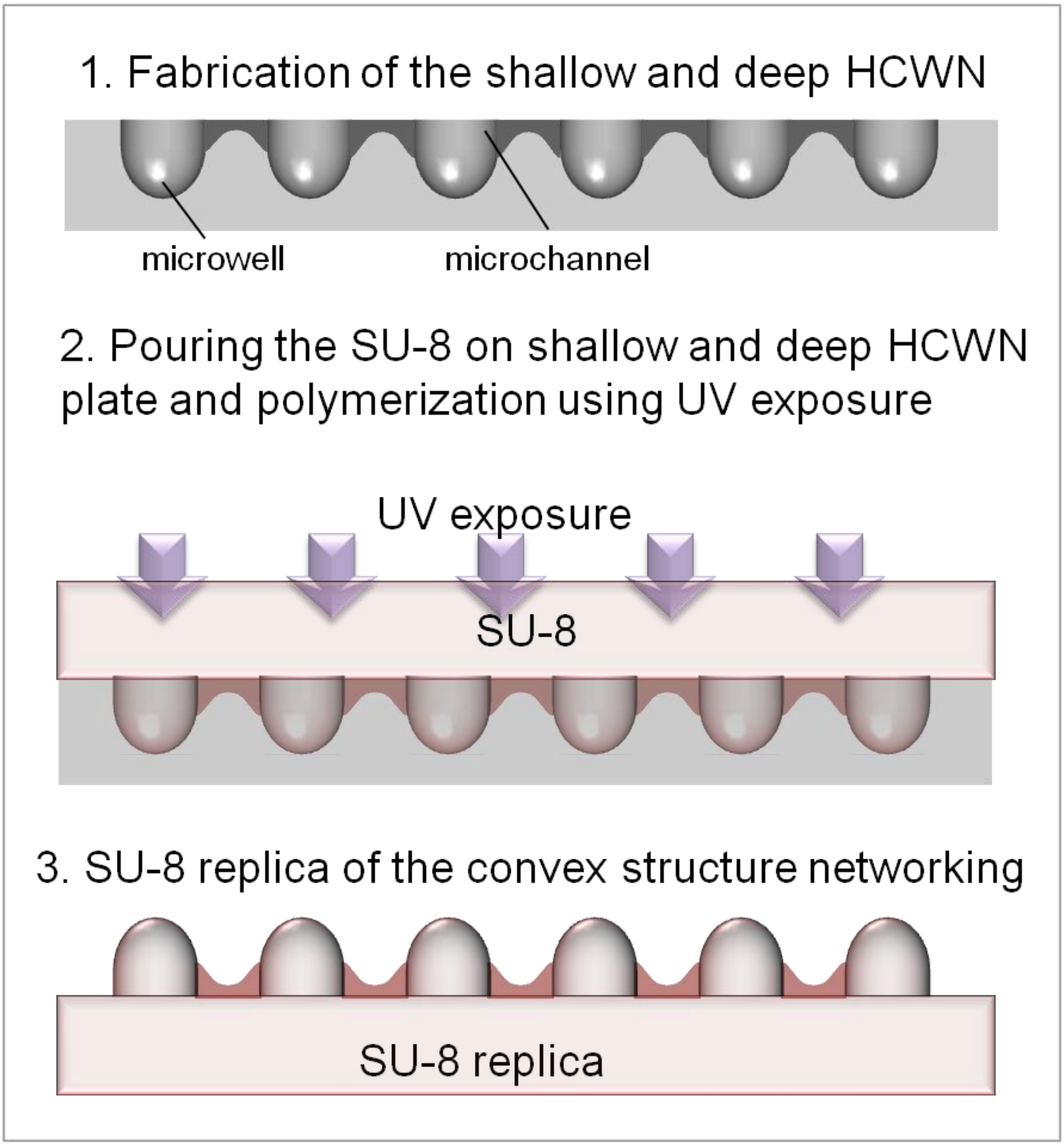

Fig. S2. Schematic diagram of the SU-8 replica fabrication process for shallow and deep HCWN plates. The following changes to figure text are suggested: 1. Fabrication of shallow and deep HCWN plates. 2. Pouring SU-8 n shall and deep HCWN plates3. Su-8 replica of the convex structure network.

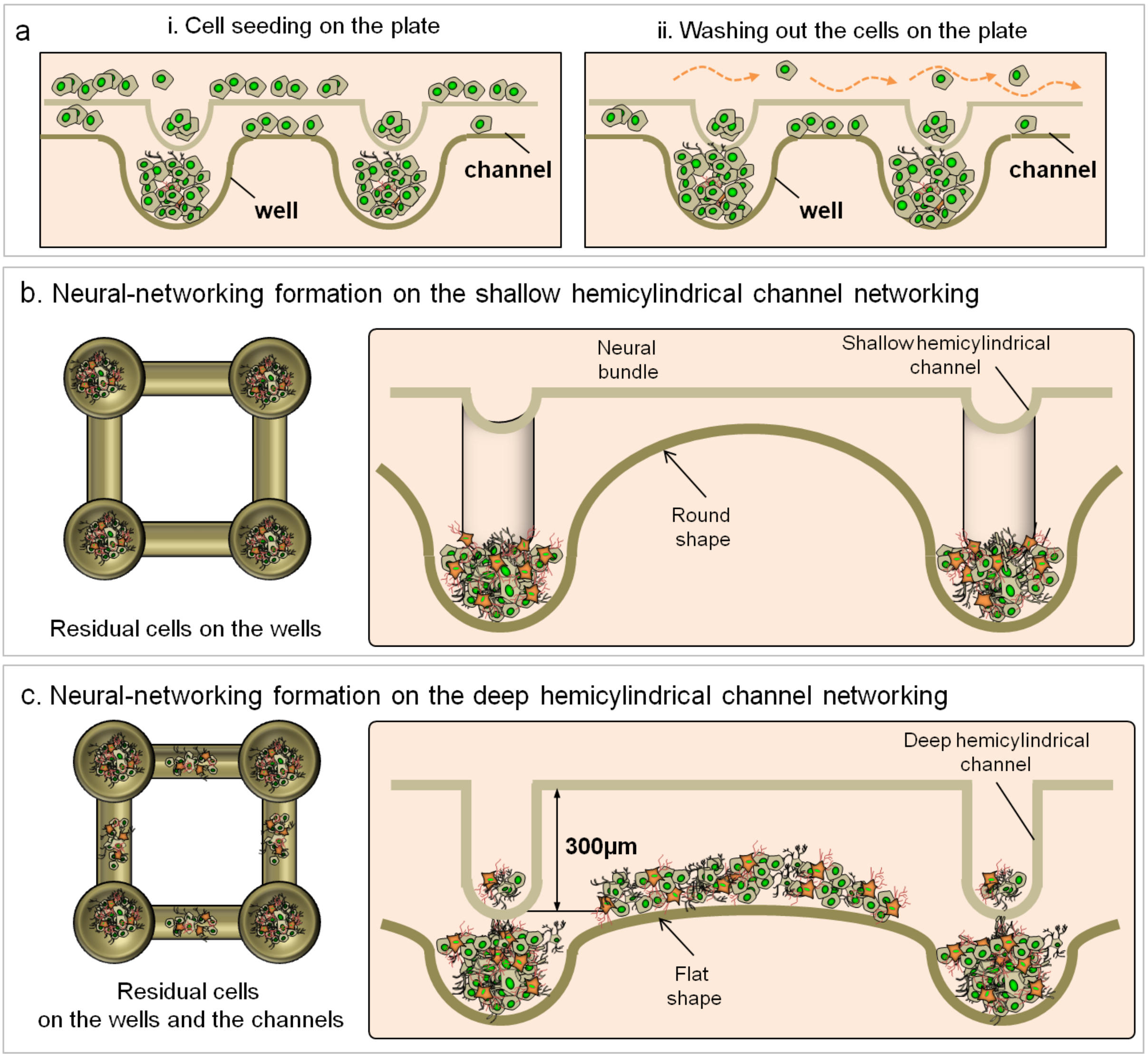

Fig. S3. Cell seeding on shallow and deep HCWN plates. (a) A 1-ml cell suspension (2.0 × 10^9 cells/ml) was directly seeded onto shallow and deep HCWN plates with repeated gentle pipetting. Ten minutes after seeding, neural cells had settled within concave microwells and hemicylindrical channel networks (i); culture medium was then gently applied to remove cells that did not settle (ii). (b, c) Settled cells after the wash-out process in the shallow HCWN system (b) and deep HCWN system (c).

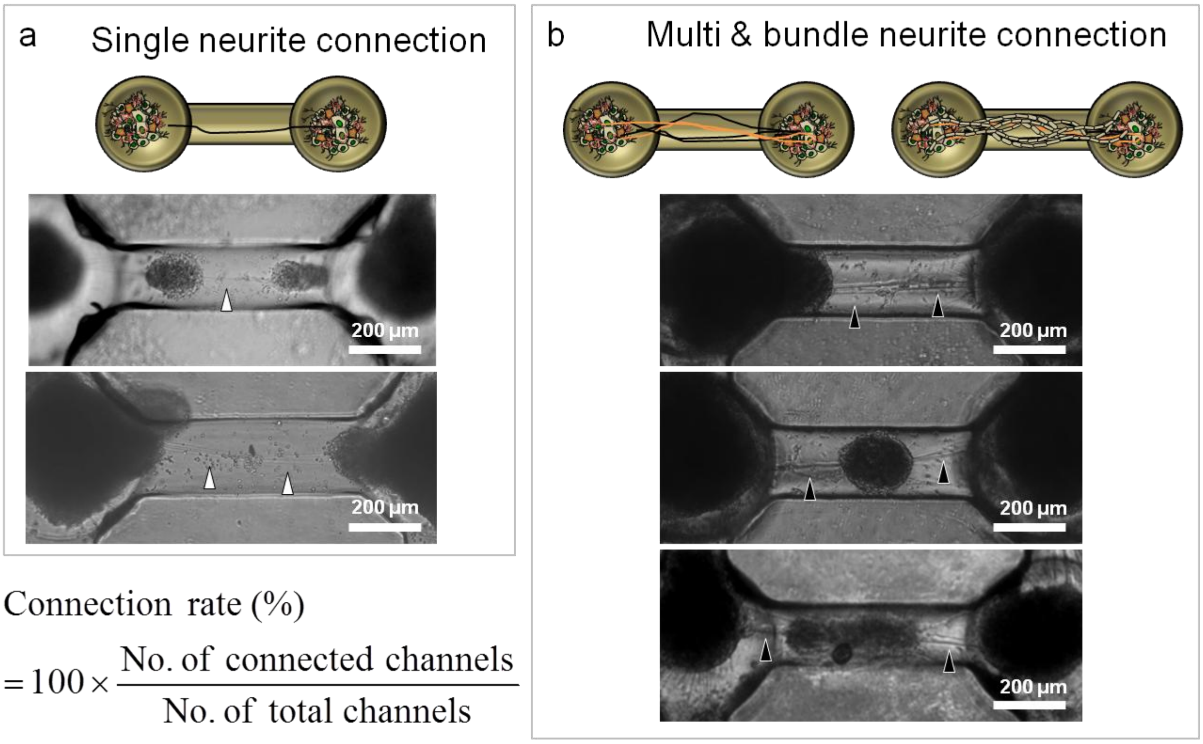

Fig. S4. Definition of neurite connection. (a) Single neurite connection through the channel. (b) Multi-connection and bundled neurite networks through the channel.

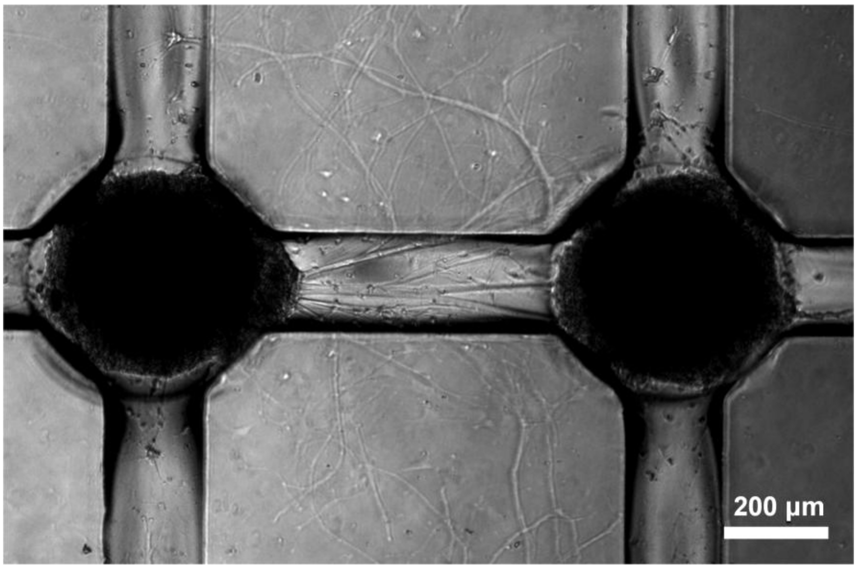

Fig. S5. Neurites growing outside the channel in the shallow HCWN system.
